# Oral Health Status of Patients with Lysosomal Storage Diseases in Poland

**DOI:** 10.3390/ijerph14030281

**Published:** 2017-03-09

**Authors:** Damian Drążewski, Małgorzata Grzymisławska, Katarzyna Korybalska, Natasza Czepulis, Marian Grzymisławski, Janusz Witowski, Anna Surdacka

**Affiliations:** 1 Department of Conservative Dentistry and Periodontology, Poznan University of Medical Sciences, 60-812 Poznan, Poland; damian.stom@gmail.com (D.D.); annasurd@gmail.com (A.S.); 2 Department of Anatomy, Poznan University of Medical Sciences, 61-781 Poznan, Poland; malgorzatagrzymislawska@ump.edu.pl; 3 Department of Pathophysiology, Poznan University of Medical Sciences, 60-806 Poznan, Poland; koryb@amp.edu.pl (K.K.); czepulis@ump.edu.pl (N.C.); 4 Department of Metabolic Disease, Nutrition and Internal Medicine, Poznan University of Medical Sciences, 60-355 Poznan, Poland; mariangrzym@ump.edu.pl

**Keywords:** lysosomal storage disease, oral health, developmental disabilities, saliva

## Abstract

Patients with lysosomal storage diseases (LSDs) suffer from physical and mental disabilities, which together with poor access to professional care may lead to impaired oral health. This cross-sectional case-control study characterized the status of oral health in patients with LSDs in Poland. Thirty-six children and young adults with various forms of LSDs were examined. The data were compared with those from age- and sex-matched healthy controls. Exemplary cases were presented to highlight typical problems in oral care associated with LSDs. When possible, saliva was collected and analyzed for total protein, inflammatory mediators, and antioxidant status. Generally, patients with LSDs had significantly higher prevalence of caries, inferior gingival status, and inadequate oral hygiene. The severity of oral health impairment in mucopolysaccaridoses, the most common LSD in Poland, was similar to that seen in patients with mannosidoses or Pompe disease. Saliva could be collected only from few less handicapped patients. In MPS, it did not appear to differ significantly from the controls, but in patients with Pompe disease it contained lower concentrations of vascular endothelial growth factor (VEGF) and monocyte chemoattractant protein-1 (MCP-1), but higher levels of tumor necrosis factor receptors 1 and 2 (TNF-R1, TNF-R2) and myeloperoxidase (MPO). In conclusion, Polish patients with LSDs have an inadequate level of oral hygiene and substantially deteriorated oral health.

## 1. Introduction

Lysosomal storage diseases (LSDs) are a heterogeneous group of rare inherited metabolic disorders characterized by defective activity of lysosomal enzymes [1]. A lysosomal enzyme deficiency results in an impaired degradation of macromolecules and the abnormal accumulation of un-cleaved substrates. These processes lead ultimately to progressive cellular damage and severe organ malfunction. Although various LSDs share many clinical features, they also differ significantly in terms of associated disability. Patients with LSDs often have altered craniofacial features and skeletal and dental abnormalities [2]. The latter include developmental alterations both in teeth and in bone support.

Given the rarity of LSDs, the scientific literature on dental status is scarce and limited to sporadic case reports. The best documented studies include the analysis of oral manifestations in cohorts of 12, 17, and 46 patients with mucopolysaccharidoses (MPS) in Brazil, Asia, and the UK, respectively [2,3,4]; in 82 patients with aspartylglucosaminuria in Finland [5]; and in 87 patients with Gaucher disease in Israel [6]. Detailed records of MPS-associated dental abnormalities can be found both in earlier extensive reviews [4,7] and in more recent original reports [8,9,10,11,12,13,14].

MPS represent the largest group of LSDs in Poland with an estimated birth prevalence of 1.81 per 100,000 [15]. A recent epidemiological survey revealed that five types of MPS had been diagnosed in Poland over the past four decades in a total of 392 individuals [15]. Other LSDs are less prevalent and some of them occur only as anecdotal cases. The Society for Mucopolysaccharidoses and Rare Diseases (SMRD) is a leading non-governmental organization dedicated to helping people with LSDs in Poland (http://chorobyrzadkie.pl). Established in 1990, the SMRD works to provide information and support for patients and families as well as increase public and professional awareness. During conferences held regularly by the SMRD, it has transpired that oral health in Polish LSD patients is a rather neglected issue, the maintenance of oral hygiene often poses a problem for caregivers, and the access to specialized dental care is being reported as difficult.

While it is recognized that patients with MPS have increased oral health needs [4,16], the degree to which these needs are met depends largely on socio-economic factors that differ across the countries. In this respect, it has recently been reported that the level of oral hygiene in patients with MPS in the UK was surprisingly similar to that in the general population [4]. We have hypothesized that this may not be a case in Poland and have therefore set out to examine the status of oral health in Polish patients with LSDs in comparison with the general population.

## 2. Methodology

This was a cross-sectional case-control investigation that involved 36 individuals with LSDs. These patients participated in rehabilitation training courses in the SMRD center. An informed consent was obtained either from patients or from their legal guardians. The study was conducted in accordance with the Declaration of Helsinki and approved by the Bioethics Committee of the Poznan University of Medical Sciences (decision 1020/13). Patient characteristics are summarized in Table 1.

The patients represented all types of mucopolysaccharidoses found in Poland, as well as other rare LSDs. The teeth were assessed as is, i.e., without prior drying and/or cleaning. The dental status was expressed as the DMFT index, which is the total number of teeth decayed, missing, or filled. The scoring ranges from 0 to 20 for deciduous teeth (dmft) and from 0 to 28 for permanent teeth (DMFT). Oral hygiene status was assessed by measuring the Plaque Index (PL-I, ranged 0–3), and the simplified oral hygiene index (OHI-s, ranged 0–6). Gingival status was expressed as the Gingival Index (GI, ranged 0–3). The indices were calculated from the following teeth: 11, 16, 26, 31, 36, and 46 (if a given tooth was missing, the adjacent tooth was assessed). For comparison, oral health was examined in healthy age- and sex-matched individuals who were undergoing routine dental check-up (see below for details).

Unstimulated whole mixed saliva was collected as is by asking the patient to spit into a sterile container. It could be obtained from only 12 patients who agreed and were able to do so. Samples were centrifuged to remove any debris, aliquoted and stored at −80 °C until assayed. Saliva was measured for total antioxidant status (TAS) with a TAS kit (Randox Laboratories, Crumlin, UK) and for myeloperoxidase (MPO), monocyte chemoattractant protein-1 (MCP-1), tumor necrosis factor receptors 1 and 2 (TNF-R1 and TNFR2), vascular endothelial growth factor (VEGF), soluble intercellular adhesion molecule-1 (sICAM-1) and matrix metalloproteinase-2 (MMP-2) using DuoSet^®^ Immunoassays (R&D Systems, Minneapolis, MN, USA). All assays were performed as per manufacturers’ instructions. Given the patients’ status, it was impossible to standardize the procedure of saliva collection with regard to its timing, duration, and flow rate. Therefore, the analyte concentrations were normalized by protein content. The protein levels were measured with the Bradford method using Protein Assay Dye Reagent (Bio-Rad, Munich, Germany).

Statistical analysis was performed using GraphPad Prism™ 6.05 software (GraphPad, La Jolla, CA, USA). The data were analyzed with the Mann–Whitney U test, analysis of variance (Anova) and Spearman correlation, as appropriate. A *p* value < 0.05 was considered significant. The data are presented as means and standard deviations (SDs). 

## 3. Results

### 3.1. Oral Examination

Since the group of patients turned out to be very heterogeneous in terms of demographic criteria, staging of the disease, the presence of mental retardation, and the degree of disability, the data on their oral health status were presented as anonymized individual records (Table 1). Detailed comparisons between various LSDs and between LSDs and the controls were complicated by the fact that some LSDs were represented only by very few (or even single) patients and that the patients differed significantly in age. The latter aspect was of importance since a preliminary analysis of healthy individuals revealed that several parameters correlated clearly with age. Therefore, we chose to compare three groups of patients: with mucopolysaccharidoses (all types), alpha-mannosidosis, and with Pompe disease. For each of these categories, a precisely age-matched control group was selected and the differences were recorded as a percentage of control values. These fractional values were used to compare the three sub-groups of LSD patients.

**Figure 1:**
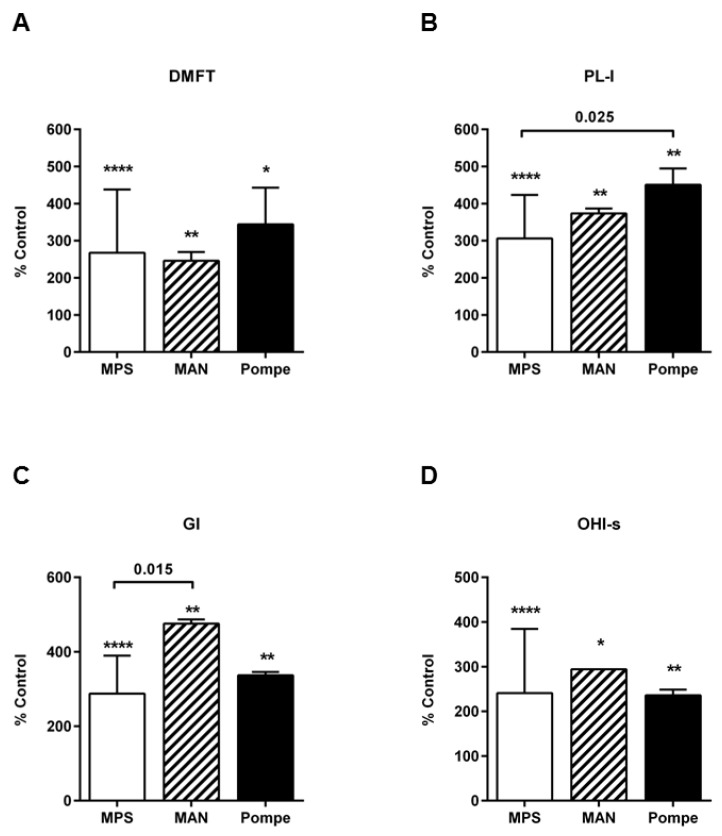
Parameters of oral health status in patients with different lysosomal storage diseases (LSDs): (**A**) the number of teeth decayed, missing, or filled (DMFT); (**B**) Plaque Index (PL-I); (**C**) Gingival Index (GI); and (**D**) simplified Oral Hygiene Index (OHI-s). DMTF score reflects combined data for permanent and deciduous dentition. The data were derived from 24 patients with mucopolysaccaridoses (MPS), four patients with mannosidosis (MAN), and three patients with Pompe disease, and compared with appropriate age-matched controls (*n* = 20, 9, and 10 for MPS, MAN, and Pompe disease, respectively). The results are presented as means ± SD. Asterisks represent significant differences compared with the controls (* *p* < 0.05; ** *p* < 0.01; *** *p* < 0.001; **** *p* < 0.0001).

On the whole, patients with LSDs had significantly higher prevalence of caries, poorer gingival status, and inadequate oral hygiene, as assessed by DMFT, GI, PL-I, and OHI-s scores, respectively. These scores in LSD patients were approximately 2–5-fold higher than in the controls. There were no major differences in DMFT and OHI-s among the three different categories of LSDs (Figure 1). However, patients with MPS seemed to have lower PL-I and GI scores than patients with mannosidosis or with Pompe disease.

### 3.2. Exemplary Cases

The case reports presented below highlight typical problems encountered in patients representing three main types of LSDs analyzed.

#### 3.2.1. Case 1. A 17-Year Old Boy with MPS Type IIIA (Patient No. 8 in Table 1)

This patient’s development seemed to be normal for the first 8–9 years. At this stage, the parents noticed a significant change in the boy’s behavior, who became hyperactive, anti-social and aggressive. The diagnosis of Sanfilippo syndrome was made at the age of 10 years. The disease progressed and at the age of 14 years the boy stopped talking and responding to orders. He developed severe contractures in all extremities and became unable to walk unassisted. Nutrition required a feeding tube. The only oral hygiene measure that was possible to apply was gentle mopping with a swab. At the age of 15, the patient underwent a previous (and the only) dental check-up with subsequent tooth extractions and treatment. All procedures were performed under general anesthesia.

The examination of oral cavity was difficult and possible only in the presence and with the assistance of the child’s mother. The DMFT-index was 22 with eight carious teeth, 10 teeth missing and four teeth filled. All teeth and interdental spaces were covered extensively with plaque and tartar along the gum line. The gingivae were reddened, swollen and bled easily upon light touch. The swallowing reflex was abolished resulting in permanent drooling and angular cheilitis.

#### 3.2.2. Case 2. A 24-Year Old Man with Alpha-Mannosidosis (Patient No. 31 in Table 1)

The patient had thickened facial features, a prominent forehead with a low hairline, a flattened nasal bridge, rounded eyebrows, and enlarged ear auricles. He suffered from severe mental retardation and restlessness, which made oral inspection very difficult. The examination revealed a significant macroglossa, prognathism, caries, and an extensive presence of plaque and tartar. The oral hygiene indices were PL-I = 3.0 and OHI-s = 5. There was evident gingivitis and gingival, palatal, and buccal hyperplasia. Few months after the examination the patient died.

#### 3.2.3. Case 3. A 17-Year Old Boy with Late-Onset Pompe Disease (Patient No. 25 in Table 1)

The patient had evident prognathism, malocclusion, and taurodondism of the jaw molars. He had an extensive build-up of plaque and tartar and poor oral hygiene indices. The patient was not mentally affected but suffered from progressive muscle weakness, including upper extremities, which made it difficult for him to use toothbrush and perform normal oral hygiene regimens.

### 3.3. Saliva Analysis

**Figure 2:**
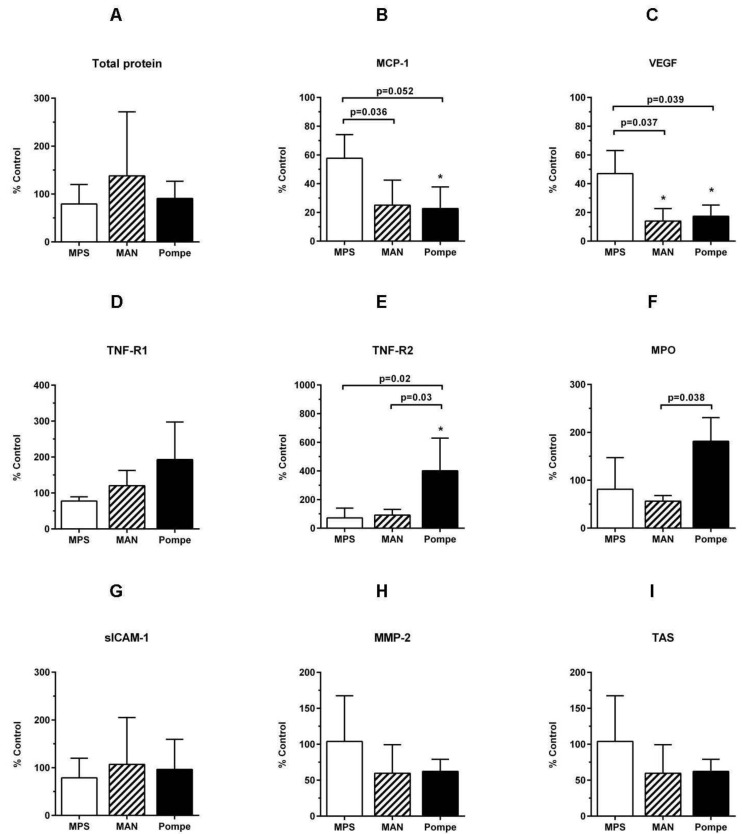
Comparison of salivary concentrations of selected mediators. Saliva was collected from patients with mucopolysaccharidoses (MPS), mannosidosi (MAN) and Pompe disease (three patients from each group, as indicated in Table 1) and age-matched healthy individuals undergoing routine dental examination. The data are presented as percentages (means ± SDs) of control values.

Samples of the saliva could be collected only from few patients, including nine patients from the three main subgroups examined (Table 1). The concentrations of analyzed parameters were normalized per salivary protein contents and compared with appropriate age-matched control groups. Saliva from MPS patients did not appear to differ significantly from the controls (Figure 2). However, patients with Pompe disease and mannosidosis had lower salivary concentrations of VEGF and MCP-1. Pompe disease was also associated with higher salivary concentrations of TNF-R2, TNF-R1 and MPO.

## 4. Discussion

The main observation of the study was that of poor oral hygiene, high prevalence of caries and gingivitis, and significant loss of teeth in patients with rare inherited metabolic diseases. The study clearly exemplified the unmet dental needs in people with developmental disabilities. The problem is gaining increasing recognition among health care professionals as the advances in medicine make it more likely for the affected children to survive into adulthood. In this respect, our observations entirely support the implications of recent reviews on oral health in handicapped children [4,16].

Poor oral health in LSDs has multiple causes. First, many LSDs lead with time to cognitive and physical impairment, which makes it difficult or even impossible for patients to follow hygiene recommendations. Indeed, our observations confirm that the greatest deterioration of oral health is seen in severely disabled patients with a long course of the disease. Dysphagia that often develops in LSDs requires administration of puréed foodstuffs that easily adhere to dental surfaces and accelerate caries development [17,18]. Such products are usually rich in carbohydrates and poor in coarse elements, which exacerbates their adverse impact on oral health [19,20]. LSD patients suffer also from retching, choking and gastro-esophageal reflux with regurgitation of the gastric contents into the mouth. Exposure to gastric acid may damage enamel surfaces and thus facilitate caries progression [21]. Advanced stages of LSDs may also be associated with hypersensitivity to smell, taste, or color of the food, which reduces dietary options and may lead to nutritional deficiencies. Deficiencies in vitamin A and C may result in increased susceptibility to infections and gingival and/or periodontal inflammation [22]. Furthermore, LSDs may lead to abnormalities in facial skeleton and tooth structure, including cleft lift and palate, missing, abnormal or extra teeth, and enamel or dentin hypoplasia [23]. Patients may also suffer from bruxism resulting in occlusal trauma [24] and gingival recession [25]. The patients in advanced stages of LSDs often require nutrition through gastrostomy or feeding tubes. Bypassing the oral cavity eliminates biting and chewing that normally contribute to oral health by scrubbing the teeth and by stimulating saliva secretion. Saliva release may further be interfered with by medication used to manage neurological complications.

Saliva is of paramount importance for the maintenance of oral homeostasis. In the present study, we have explored whether it is feasible to collect saliva from LSD patients and whether its analysis may provide some information on the patients’ oral health status. It turned out that the samples could be collected only from few patients, less affected by the disease. Even then, the collection procedure was difficult to standardize and the saliva flow rates were impossible to calculate. Therefore, the samples were collected as is, the analysis was limited to the mediators known to be detectable in saliva [26] and their concentrations were normalized per total salivary protein contents. Nevertheless, the interpretation of the data thus obtained is challenging and may be prone to bias.

The consequence of decreased salivary concentrations of VEGF in some LSDs is unclear. It appears that salivary VEGF is a key mediator of oral mucosa wound healing [27]. Thus, low levels of VEGF may predispose LSD patients to delayed tissue repair and protracted inflammation. It has also been demonstrated that endothelial cell dysfunction seen in mucopolysaccharidosis type VI may be related to reduced VEGF expression [28]. On the other hand, it has recently been demonstrated that VEGF levels can be significantly increased in cerebrospinal fluid of patients with MPS type I [29]. This effect was interpreted as a result of disease-associated inflammation. Unfortunately, in our study we were unable to secure a sample of saliva from a patient with MPS type I.

Increased concentrations of TNF-R1, TNF-R2, and MPO in patients with Pompe disease may point to on-going inflammation in the oral cavity. In this respect, it has been suggested that chronic inflammation may contribute to gingival overgrowth seen in Pompe disease [30]. The role of salivary MPO in oral host defense is well appreciated [31]. Thus, increased levels of MPO in saliva may be viewed as a protective response to insults to oral homeostasis [32]. Indeed, increased MPO has been shown to promote the degradation of toxic lysosomal deposits [33]. On the other hand, however, a chronically increased MPO activity may lead to lysosomal stress and cell death [33].

As the degree of disability increases with time, the LSD patients become increasingly dependent on their families and/or caregivers for regular oral hygiene. Our observations indicate that the dedication of family members to the task is enormous. However, they are often overwhelmed by other medical issues affecting the children, so that they may require coordinated professional support tailored specifically to patient’s needs. When implemented early enough, such measures may help prevent, or at least, slow down the deterioration of oral health. However, we found out that the access to such a specialized dental care in Poland may be limited because of reimbursement issues and the lack of dental professionals qualified to deal with disabled patients. These observations may account for the differences across the countries in oral health status of LSD patients [4].

## 5. Conclusions

In conclusion, patients with LSDs may suffer from poor dental and gingival status, and inadequate oral hygiene. The severity of these problems is generally proportional to the degree of disability associated. While salivary concentrations of several inflammatory mediators may show some differences of pathophysiological significance compared to the general population, the sampling of saliva is difficult both to perform and standardize. Therefore, the value of saliva as a diagnostic medium in this patient group may be limited. Our observations confirm that LSD patients constitute a population with special needs in terms of oral health and require a coordinated oral care.

## Figures and Tables

**Table 1:** Basic demographic and oral health parameters in patients with different lysosomal storage diseases.

Patient No.	Sex	Age	Mental Retardation	The Disease (Eponym)	The Enzyme Lacking	Dentition	DMFT	dmft	PLI	OHI-s	GI	Saliva Sample
1	M	19	N	Mucopolysaccharidosis I (Hurler syndrome)	Alpha-L-Iduronidase	P	4	-	0.7	1	0.75	N
2	M	10	Y	Mucopolysaccharidosis II (Hunter syndrome)	Iduronate sulphatase	P	8	-	1.6	2	1.58	Y
3	M	10	Y			P	9	-	2.5	4	2.04	Y
4	M	30	N			P	21	-	3.0	6	3.00	N
5	M	7	Y			M	4	7	2.1	2	1.91	N
6	M	12	Y			P	9	-	2.0	2	1.75	N
7	M	10	Y			P	8	-	0.8	1	0.95	N
8	M	17	N	Mucopolysaccharidosis IIIA (Sanfilippo syndrome A)	Heparan sulfamidase	P	22	-	2.6	-	2.37	N
9	F	8	Y			D	-	20	1.8	2	1.58	N
10	M	8	Y			P	16	-	2.8	4	2.37	N
11	F	7	Y			M	8	4	1.7	2	1.54	N
12	F	13	Y			P	7	-	1.7	2	1.41	N
13	F	9	Y			M	4	4	1.8	2	1.30	N
14	M	10	Y	Mucopolysaccharidosis IIIB (Sanfilippo syndrome A)	*N*-acetyl-alpha-D-glucosaminidase	M	4	4	1.8	2	1.30	N
15	F	16	Y			P	9	-	2.0	2	1.91	N
16	F	14	Y			P	4	-	0.8	1	0.87	N
17	F	8	Y			M	4	8	1.8	2	1.54	N
18	F	12	Y			P	11	-	2.8	3	2.33	N
19	M	16	Y			P	8	-	1.7	3	1.87	N
20	F	25	N	Mucopolysaccharidosis IVA (Sanfilippo syndrome B)	Galactose-6-sulfate sulfatase	P	8	-	1.7	2	1.58	Y
21	M	10	Y			M	3	9	2.0	2	1.79	N
22	M	15	Y			P	7	-	2.7	4	2.54	N
23	M	30	N	Mucopolysaccharidosis IVB (Morquio syndrome B)	Beta-galactosidase	P	28	-	3.0	6	3.00	N
24	M	12	Y	Mucopolysaccharidosis VI (Maroteaux-Lamy syndrome)	Aryl-sulfatase B	P	3	-	0.9	1	0.87	N
25	M	17	Y	Pompe Disease (Glycogen storage disease type II)	Acid alpha-glucosidase	P	8	-	2.8	4	2.75	Y
26	F	14	Y			P	10	-	2.7	4	2.84	Y
27	M	12	Y			P	14	-	3.0	4	2.90	Y
28	F	23	Y	Alpha-mannosidosis	Alpha-D-mannosidase	P	14	-	3.0	5	2.85	Y
29	M	22	Y			P	14	-	3.0	5	3.00	Y
30	F	18	Y			P	15	-	2.8	5	2.94	Y
31	M	24	Y			P	17	-	3.0	5	3.00	N
32	M	29	Y	Niemann-Pick disease	Acid-sphingo-myelinase	P	16	-	3.0	4	3.00	N
33	M	14	Y			P	10	-	3.0	2	3.00	N
34	M	24	Y	Gangliosidosis I	Beta-galactosidase	P	14	-	3.0	5	2.85	Y
35	M	11	Y	Mucolipidosis II (Inclucion-cell disease)	Phosphotransfe-rase	M	5	6	3.0	4	3.00	Y
36	F	24	Y	Metachromatic leukodystrophy (MLD)	Aryl-sulfatase A	P	9	-	1.8	3	2.90	Y

Dentition: P, permanent; D, deciduous; M, mixed.
